# A New Putative Caulimoviridae Genus Discovered through Air Metagenomics

**DOI:** 10.1128/MRA.00955-18

**Published:** 2018-10-11

**Authors:** Alberto Rastrojo, Andrés Núñez, Diego A. Moreno, Antonio Alcamí

**Affiliations:** a Centro de Biología Molecular Severo Ochoa, Consejo Superior de Investigaciones Científicas and Universidad Autónoma de Madrid (CSIC-UAM), Madrid, Spain; b Escuela Técnica Superior de Ingenieros Industriales, Universidad Politécnica de Madrid (ETSII-UPM), Madrid, Spain; University of Arizona

## Abstract

Members of the Caulimoviridae family are important plant pathogens. These circular double-stranded DNA viruses may integrate into the host genome, although this integration is not required for the viral replication cycle. Here, we describe three complete genomes belonging to a new putative Caulimoviridae genus discovered through air metagenomics.

## Abstract (precis)

Members of the Caulimoviridae family are important plant pathogens. These circular double-stranded DNA viruses may integrate into the host genome, although this integration is not required for the viral replication cycle.

## ANNOUNCEMENT

Caulimoviridae is the only known double-stranded DNA (dsDNA) virus family infecting plants that replicates by reverse transcription (1). Members of this family have circular genomes of 7,000 to 8,200 bp with discontinuities in both strands coding for 1 to 8 open reading frames (ORFs) (2). The replication cycle is episomal and does not require integration into the host genome. However, integration can occur during the nonhomologous end-joining repair of dsDNA breaks in host genomes, leaving a fingerprint of past infections (3). The study of these endogenous viral elements suggests that this family would have emerged approximately 320 million years ago (4).

Here, we describe three complete viral genomes belonging to the family Caulimoviridae. These genomes were obtained from air samples collected in Madrid, Spain (40.439881°N, 3.689409°W) using different devices, namely, a Hirst spore trap, a Surface Air System DUO 360 instrument, and a Burkard multivial cyclone sampler (our unpublished data). A PowerSoil DNA isolation kit was used to extract total DNA. Samples were then sequenced using Illumina technology, obtaining 40 million paired-end reads for each sample (2 × 125 nucleotides). Raw reads were quality filtered using PRINSEQ (mean quality score of 25 and length of >75) (5), assembled with IDBA_UD with default parameters (6), and classified using a BLAST search against the NCBI nonredundant database (e-value, <1e-3; score, >50) (7). Three contigs of ∼7 kb were assigned to the family Caulimoviridae and were circularized using Minimus2 (8). These viral genomes are 98.8 to 99.3% identical to each other and therefore belong to the same species. All three genomes have a minus-strand primer binding site, a polypurine tract, and polyadenylation signals, and they have only one large ORF coding a 2,129-amino acid polyprotein with several domains, movement protein (amino acids [aa] 57 to 222), coat protein (aa 602 to 838), aspartic protease (aa 991 to 1124), reverse transcriptase (aa 1144 to 1619), and RNase H (aa 1627 to 1740) (9). All of these features are characteristic of members of the genus Petuvirus (2). However, the reverse transcriptase (RT)-RNase H domain shares only 40% identity with Petunia vein clearing virus (PVCV), the unique member of the genus Petuvirus.

By searching against the complete or near-complete endogenous viral genomes described by Diop et al. (4), we were able to find the closest relative, Pinus taeda gymnendovirus 2 (PtGy2), which shares a 77% identity at the nucleotide level with the three new genomes. PtGy2 contains five ORFs, but the rearrangement the ORFs generated a single ORF coding a 2,106-aa polyprotein that is 79% identical to the proteins of the newly discovered viruses. This identity increased to 84% when the RT-RNase H domain was examined. Therefore, the new genomes could represent a replication competent version of the PtGy2 endogenous element, which could have been fragmented because of the accumulation of several mutations or indels due to its integration in the genome of Pinus taeda. Interestingly, ∼80% of the shotgun metagenomic reads were assigned to Pinus taeda. Additionally, we were able to detect by PCR these new viruses in a Pinus nigra sample from the vicinity of where the air samples were collected, suggesting that these new viruses are likely Pinus pathogens. The phylogenetic analysis (10, 11) of the RT-RNase H protein of representative Caulimoviridae members showed a strong relation of the new viruses with PtGy2 and with Picea glauca gymnendovirus 2, another endogenous element, all of them forming an ancient branch in clade B, to which PVCV belongs (4). In conclusion, we propose that these new genomes may represent a new genus of Caulimoviridae-infecting gymnosperms, in contrast to Petuvirus-infecting angiosperms, which could represent the replicative counterpart of the endogenous Gymnendovirus 2 genus recently described.

### Data availability.

The three complete genome sequences reported here have been deposited in GenBank under the accession numbers MH551471, MH551472, and MH551473. Shotgun raw reads have also been deposited in the European Nucleotide Archive (ENA) under the accession numbers ERX2313857, ERX2313858, and ERX2313863.
